# Potential complications of CAD/CAM-produced resin composite crowns on molars: A retrospective cohort study over four years

**DOI:** 10.1371/journal.pone.0266358

**Published:** 2022-04-07

**Authors:** Miyu Inomata, Akio Harada, Shin Kasahara, Taro Kusama, Akane Ozaki, Yusuke Katsuda, Hiroshi Egusa

**Affiliations:** 1 Division of Molecular and Regenerative Prosthodontics, Tohoku University Graduate School of Dentistry, Sendai, Japan; 2 Division for Regional Community Development, Liaison Center for Innovative Dentistry, Tohoku University Graduate School of Dentistry, Sendai, Japan; Universitat Bern, SWITZERLAND

## Abstract

**Purpose:**

Evaluation of the clinical performance of computer-aided design/computer-aided manufacturing-produced resin composite crowns (CAD/CAM composite crowns) on molars with a particular focus on placement location.

**Methods:**

A retrospective cohort study was performed based on the clinical records of patients with CAD/CAM composite crowns on molars (June 2016 to March 2021). The hazard ratios (HRs) and 95% confidence intervals (95% CIs) were estimated based the Cox proportional hazard model to evaluate the effect of tooth location on complication type and occurrence. Covariates included crown location (maxilla/mandible, distalmost tooth/not distalmost tooth, and first molar/second or third molar) and endodontically treated (nonvital) or untreated (vital) tooth.

**Results:**

Overall, 362 crowns were evaluated (mean follow-up: 378 days, median: 286 days), and 106 crowns (29.3%) showed complications, most frequently crown debonding. The cumulative success and survival rates were 70.9% and 93.7%, respectively, after 1 year and 49.5% and 86.5%, respectively, after 3 years. There was no significant difference in the HRs and log-rank tests in the Kaplan–Meier curves based on crown location parameters (*P* > 0.05). However, placement on vital teeth was associated with higher risks than on nonvital teeth (HR, 1.55; 95% CI, 1.03–2.23). In addition, the cement as a covariate yielded a high HR.

**Conclusions:**

The location of CAD/CAM composite molar crowns is unlikely a risk factor for complications; therefore, these crowns can be clinically applied to all molars. However, the application of such molar crowns to vital teeth and the use of a cement other than adhesive resin cement present risks.

## Introduction

Recently, remarkable developments in computer-aided design/computer-aided manufacturing (CAD/CAM) technologies have been made, and these have become an indispensable part of dental treatment. Although zirconia and lithium disilicate glass ceramic blocks are commonly used for CAD/CAM-produced crown treatments, hybrid-type resin composite blocks are also an option for crown treatment with a homogeneous material [1,2]. The advantages of CAD/CAM-produced resin composite crowns (CAD/CAM composite crowns) are that they cause less wear on the antagonist teeth than zirconia, especially if polishing is insufficient; further, there are no heating processes such as sintering or crystallization during the crown fabrication process, and the wear of the milling bar is less than that with other harder ceramic blocks. Thus, because the mechanical properties of CAD/CAM composite blocks have been improved significantly in recent years, CAD/CAM composite crowns has been applied to teeth in the molar region.

*In vitro* studies support the application of CAD/CAM composite crowns to molars based on their fracture strength and fatigue resistance [3–7]. In particular, even though resin composite blocks have low flexural strength, molar-form specimens have fracture strengths that are comparable to those of lithium disilicate glass ceramic crowns [4]. In addition, the fracture strength is 3–4 times higher than the bite force in compression tests, and the fracture strength of blocks is clinically applicable, as shown by fatigue testing [5]. Therefore, these *in vitro* studies suggest that CAD/CAM composite crowns are suitable for application in the molar region.

In contrast, a recent *in vitro* study has demonstrated the low strength of CAD/CAM resin composite blocks by conducting accelerated degradation tests in water, which challenges the long-term usage of CAD/CAM composite crowns in a clinical setting [8]. In addition, CAD/CAM composite crowns can result in different clinical outcomes compared to conventional metal crowns because of the difficulty of bonding the resin blocks, as well as the differences in the characteristics of the blocks used. In particular, compared to premolars, molars are assumed to have several mechanical disadvantages as a result of the greater occlusal force applied during biting and the anatomically lower crown height. Several clinical studies have reported the clinical outcomes of CAD/CAM composite crowns [9–11]. For example, Miura et al. published the results of a large retrospective study, reporting that the success and survival rates of CAD/CAM composite crowns applied to the premolar region were 71.7% and 96.4%, respectively, over three years [12]. The main complication was crown debonding within the first six months. However, few studies of the application of these crowns to the molar region have been reported to date. In addition, the sample sizes of studies involving molar CAD/CAM crowns are small.

In Japan, CAD/CAM composite crown treatment has been covered by the Japanese medical insurance system since 2014. As a result of the increasing demand for esthetic dental treatments, annual increase in the price of metals, and the low allergenicity of resins, these esthetically pleasing and economical resin crowns are increasingly used in metal-free dental treatments in Japan. However, because molars are subjected to greater occlusal force than premolars [13], Japanese medical insurance institutions have only slowly produced guidelines for the application of composite crowns to the molar region. In particular, application to the second molar and the distalmost tooth is not permitted, and application to the first molar is limited to patients who have occlusal support on every second molar [14]. Further, only patients diagnosed as having metal allergies are covered by insurance for crown treatment to all molars. However, there is no evidence to support these limitations.

Therefore, the purpose of this study was to evaluate the differences in the location of CAD/CAM composite crowns applied to molars on the clinical prognosis. Our null hypothesis is that differences in the crown location will not affect the clinical outcomes of CAD/CAM composite crowns applied to molars.

## Materials and methods

### Design and settings

This study was designed as a retrospective cohort study of CAD/CAM composite crowns placed on molars from June 2016 to March 2021 at Tohoku University Hospital (Sendai, Japan) and a collaborating private clinic (Yakushido Dental Clinic, Sendai, Japan). All dentists who participated in this study are active members of the Japan Prosthodontic Society (JPS), and the treatment of CAD/CAM composite crowns was conducted in accordance with the JPS guidelines [14].

CAD/CAM composite crowns should fulfill the following essential criteria for consideration in tooth preparation: 1.5–2.0 mm of occlusal reduction without residual sharp edges, a deep chamfer margin with an axial clearance of 1.0 mm and axial angle of 6–10°, and an axial wall with a smooth surface without any undercuts. The finish line was applied at the height of the gingival margin or above the gingival level. After the preparation of the abutment teeth, impressions were taken using silicone rubber impression materials (Examixfine, Exahiflex: GC, Tokyo, Japan), self-wetting hybrid polysiloxane impression materials (Fusion II: GC), or the agar–alginate-combined impression method (Dentloid Pro: Dentronics, Tokyo, Japan, and Aroma Fine Plus: GC). Stone models were fabricated using a dental stone (New plastone II LE: GC) or high-strength dental stone (New Fujirock: GC).

CAD/CAM composite crowns were fabricated in the dental laboratory of a university hospital or in private dental laboratories. The CAD systems used herein were Lava Scan ST (3M ESPE, Minnesota, USA), Aadva Scan D810 (GC), Shofu S-Wave D900 (Shofu, Kyoto, Japan), 3shape E1 (3shape, Copenhagen, Denmark), Cerec inLab MCXL (Dentsply Sirona, Tokyo, Japan), and Cerec inLab MCX5 (Dentsply Sirona). The CAM systems DWX-50 (Roland), Aadva Mill LW-1 (GC), and Aadva Harmony Wet (GC) were used in this study. The CAD/CAM resin blocks used were Cerasmart 300 (GC), Shofu Block HC Hard (Shofu), or KZR-CAD HR Block 3 (Yamakin, Osaka, Japan).

The fabricated CAD/CAM composite crowns were placed with adhesive resin cements [G-cem One (GC), SA luting (Kuraray Noritake Dental, Tokyo, Japan), Panavia V5 (Kuraray Noritake Dental), Resicem (Shofu), Esthecem II (Tokuyama, Tokyo, Japan), and Superbond C&B (Sun Medical, Shiga, Japan)] or resin modified glass ionomer cement [Fuji luting (GC)]. Sandblasting was performed on the inner surfaces of the crowns prior to cementation. Silane coupling treatment was used for the resin cements. After injecting the cement into the crown, the crown was placed on a tooth, and the excess cement was removed. For dual-cure resin cements, the excess cement was removed after temporary polymerization by light irradiation.

The retrospective data search and retrieval was carried out on laboratory prescriptions and medical health records. First, the laboratory prescriptions of CAD/CAM composite crowns were searched, and subjects’ data were collected retrospectively from the medical records for analysis. Exclusion criteria were: no final crown placement, no visit after crown placement, placement on a tooth after root separation or root resection, placement on a grafted tooth, and cases with insufficient medical records.

### Outcome variable

The entry point was when the crowns were cemented, and the end point was when any clinical complication occurred. In accordance with the previous reports [12,15], complications were defined on the basis of the problems related to the crown and abutment tooth. The complications that occurred in this study were crown debonding, crown fracture, crown chipping, post-and-core debonding, pulpitis, apical periodontitis, and abutment tooth fracture (post-and-core fracture and/or root fracture). Pulpitis and apical periodontitis were defined as cases with clinical symptoms (e.g., cold water pain, spontaneous pain, occlusal pain, and appearance of a fistula), for which endodontic treatments were necessary.

Cases where there were no complications and the fitting and tooth remained in a healthy condition were classified as success cases, whereas cases involving complications but where continued use was possible after simple treatment were classified as survival cases (e.g., crowns that reluted after debonding or had minor chipping but were repairable via polishing). In cases involving multiple complications simultaneously, the most serious complication was selected as the exemplar.

### Explanatory variables and covariates

The main predictors were the location of the crown (maxilla/mandible, first molar/second or third molar, and distalmost tooth or not distalmost tooth), and the endodontically treated (nonvital) or untreated (vital) tooth. We also considered possible covariates, including sex (male/female), age at crown placement (< 65 / ≥ 65), cement type (adhesive resin cement/other cement), placement on a removable partial denture (RPD) abutment tooth (or not), and characteristics of the antagonist tooth (natural, restored, removable, missing, implant, or unknown).

### Statistical analysis

The Kaplan–Meier method was used to describe the success and survival rates of the crowns, and survival and success curves were drawn for each covariate. The difference in survival time was evaluated by using the log-rank test. The Cox proportional hazard model was used to estimate the hazard ratios (HRs) and 95% confidence intervals (95% CIs). Two models were designed. In model 1, the location of the crown and the vital/nonvital tooth were included with sex and age; in model 2, the restorative condition of the antagonist teeth and the cement used were added to model 1, and the effect of the tooth location on the complication was analyzed. Statistical analysis was performed using JMP Pro 16.0 (SAS Institute, Cary NC, USA).

### Ethical issues

The study protocol was approved by the Research Ethics Committee of Tohoku University, Graduate School of Dentistry (approval number: 2018-3-32). Subject consent was obtained by an opt-out policy.

## Results

**Fig 1:**
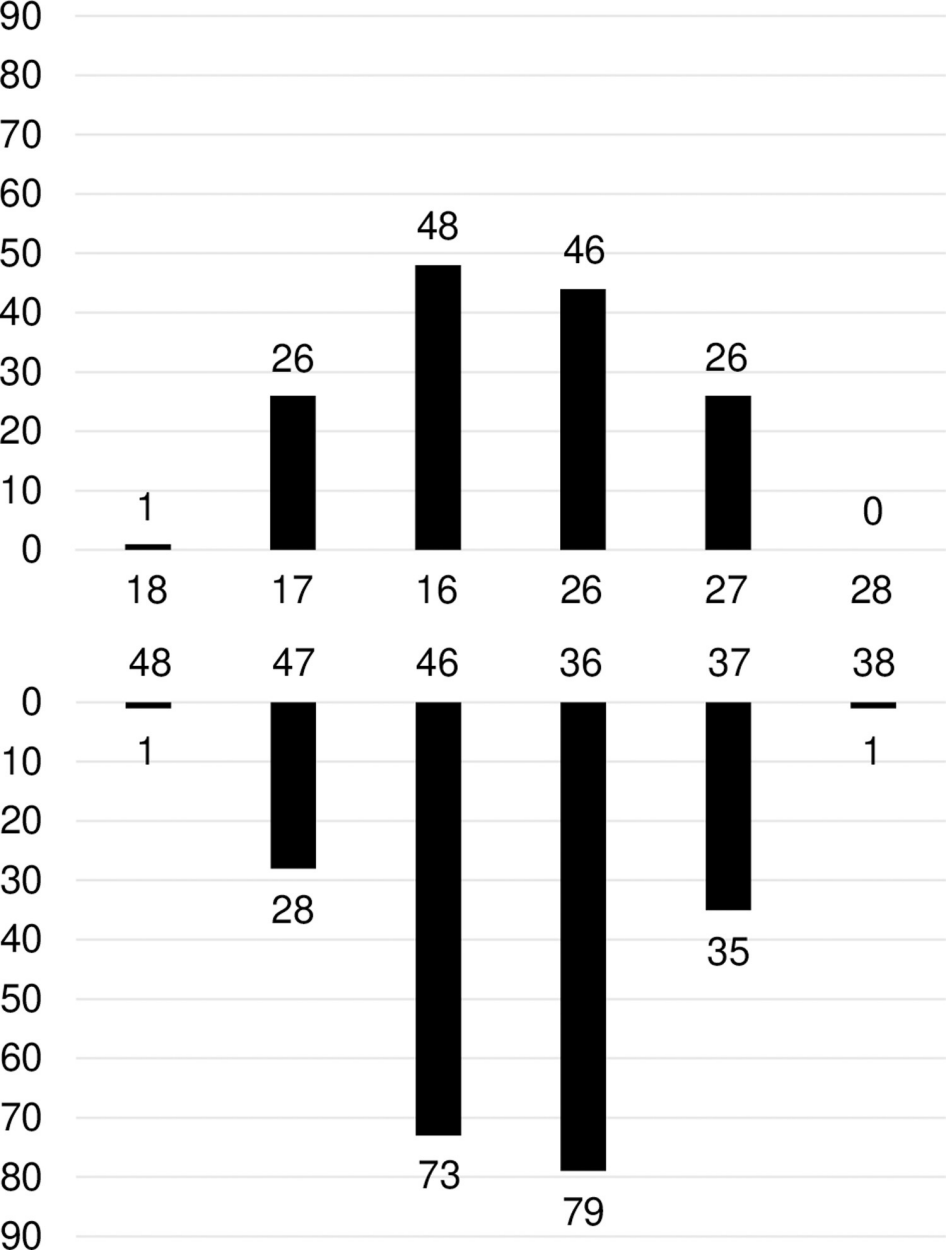
Distribution of CAD/CAM composite crowns applied to molars (*n* = 362). The tooth numbering is described using the FDI World Dental Federation notation.

A total of 402 crowns were manufactured during the observation period. Of these, 40 crowns were excluded based on the exclusion criteria. The duration of observation for the 362 crowns ranged from 6 days to 1603 days (mean, 378 days; median, 286 days). Overall, 43 of the crowns were placed on male patients and 319 on female patients. The patients were aged from 13 to 88 years with a mean age of 51.1 years [standard deviation (SD) = 14.4]. The distribution of the placement sites is shown in **Fig 1**.

**Table 1** shows the distribution of each factor regarding complications. One-hundred and twenty crowns were placed on distalmost teeth, and two crowns were placed on RPD abutment teeth. Two-hundred and sixty crowns were placed on nonvital teeth, and 102 crowns were placed on vital teeth. Two hundred seventy-five crowns were cemented with adhesive resin cement, whereas 87 crowns were fitted using other cements. Concerning the condition of the antagonist teeth, restored teeth were the most common, and implants were the least common, although some of the antagonist teeth were missing (*n* = 18).

**Table 1:** Baseline characteristics of study subjects with complications.

Category	Tooth frequency	Complication	
		No	Yes
	[*n* (%)]	[*n* (%)]	[*n* (%)]
**Overall patient numbers**	362 (100)	256 (70.7)	106 (29.3)
**Sex**			
Male	43 (11.9)	31 (72.1)	12 (27.9)
Female	319 (88.1)	225 (70.5)	94 (29.5)
**Age (years)**			
< 65	294 (81.2)	203 (69.0)	91 (31.0)
≥ 65	68 (18.8)	53 (77.9)	15 (22.1)
**Teeth**			
**Anatomical crown location**			
Maxilla	144 (39.8)	110 (76.4)	34 (23.6)
Mandible	218 (60.2)	146 (67.0)	72 (33.0)
Not distalmost tooth	242 (66.9)	170 (70.2)	72 (29.8)
Distalmost tooth	120 (33.2)	86 (71.7)	34 (28.3)
1^st^ molar	244 (67.4)	172 (70.5)	72 (29.5)
2^nd^/3^rd^ molar	118 (32.6)	84 (71.2)	34 (28.8)
**Endodontically treated tooth**			
Yes (nonvital)	260 (71.8)	192 (73.8)	68 (26.2)
No (vital)	102 (28.2)	64 (62.7)	38 (37.3)
**Cement type used**			
Adhesive resin cement	275 (76.0)	212 (77.1)	63 (22.9)
Other	87 (24.0)	44 (50.6)	43 (49.4)
**Antagonist tooth characteristics**			
Natural	81 (22.4)	54 (66.7)	27 (33.3)
Restored	244 (67.4)	173 (70.9)	71 (29.1)
Removable	14 (3.9)	11 (78.6)	3 (21.4)
Missing	18 (5.0)	14 (77.8)	4 (22.2)
Implant	3 (0.8)	3 (100)	0 (0.0)
Unknown	2 (0.6)	1 (50.0)	1 (50.0)

**Fig 2:**
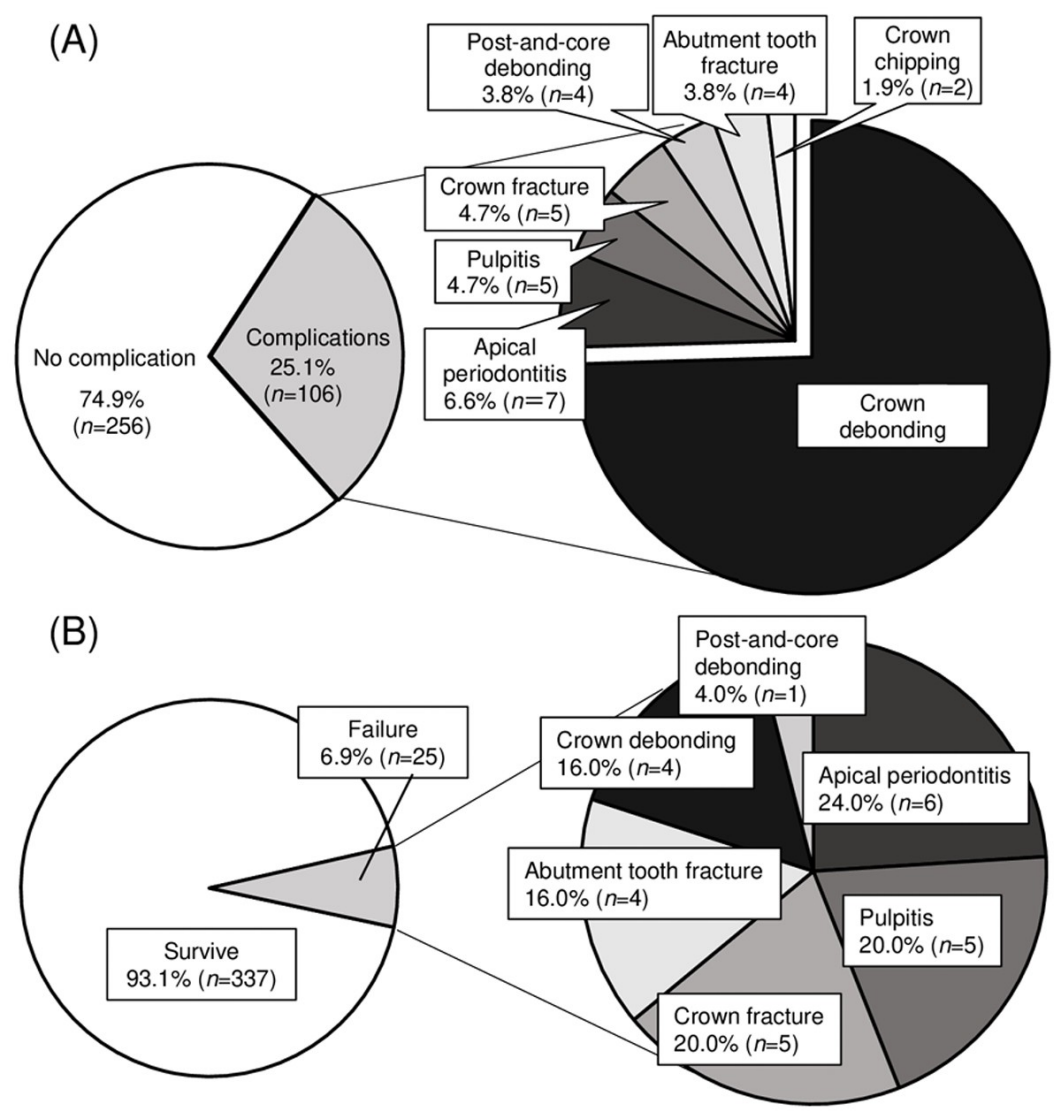
Summary and detail of complications of CAD/CAM composite crowns. (A) Types of complications occurring and (B) cause of crown failure.

A total of 106 crowns (29.3%) showed evidence of clinical problems during the observation period. **Fig 2A** shows that the most frequent complication was crown debonding (74.5%), followed by endodontic complications (apical periodontitis and pulpitis) (11.3%), crown fracture (4.7%), abutment tooth fracture (3.8%), post-and-core debonding (3.8%) and crown chipping (1.9%). A total of 25 crowns (6.9%) suffered failure (**Fig 2B**) and required either removal or tooth extraction. Endodontic complications (apical periodontitis and pulpitis), which required crown removal or open access for retreatment, was the most frequent type of failure.

**Fig 3:**
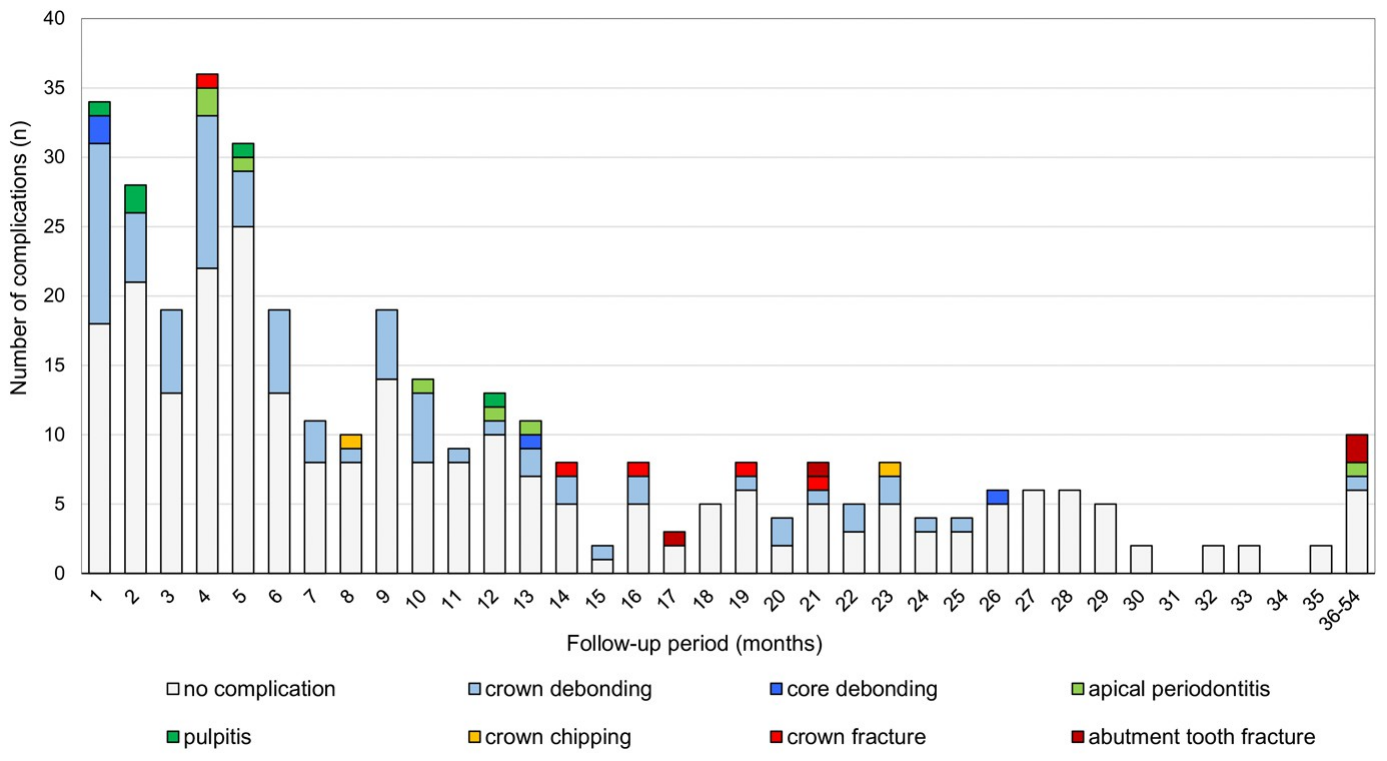
Timeline and types of complications of CAD/CAM composite crowns.

The timeline, types of complications, and success cases are shown in **Fig 3**. Crown debonding most frequently occurred in the early stages after placement and was observed over a period of more than 2 years. However, in most cases, the crown could be reluted. Pulpitis also occurred at relatively early stages, and crown fracture or abutment tooth fracture mainly occurred after one year.

**Fig 4:**
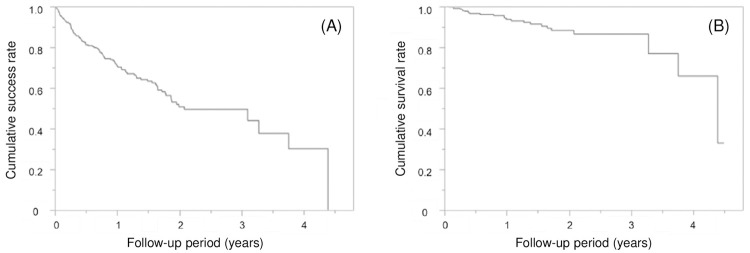
Kaplan–Meier curves showing the (A) cumulative success rate and (B) cumulative survival rate of CAD/CAM composite crowns.

The cumulative success and survival rates of the 362 CAD/CAM composite crowns were 70.9% and 93.7%, respectively, after 1 year, 50.8% and 88.3%, respectively, after 2 years, and 49.5% and 86.5%, respectively, after 3 years based on the survival curves obtained using the Kaplan–Meier method (**Fig 4**).

**Fig 5:**
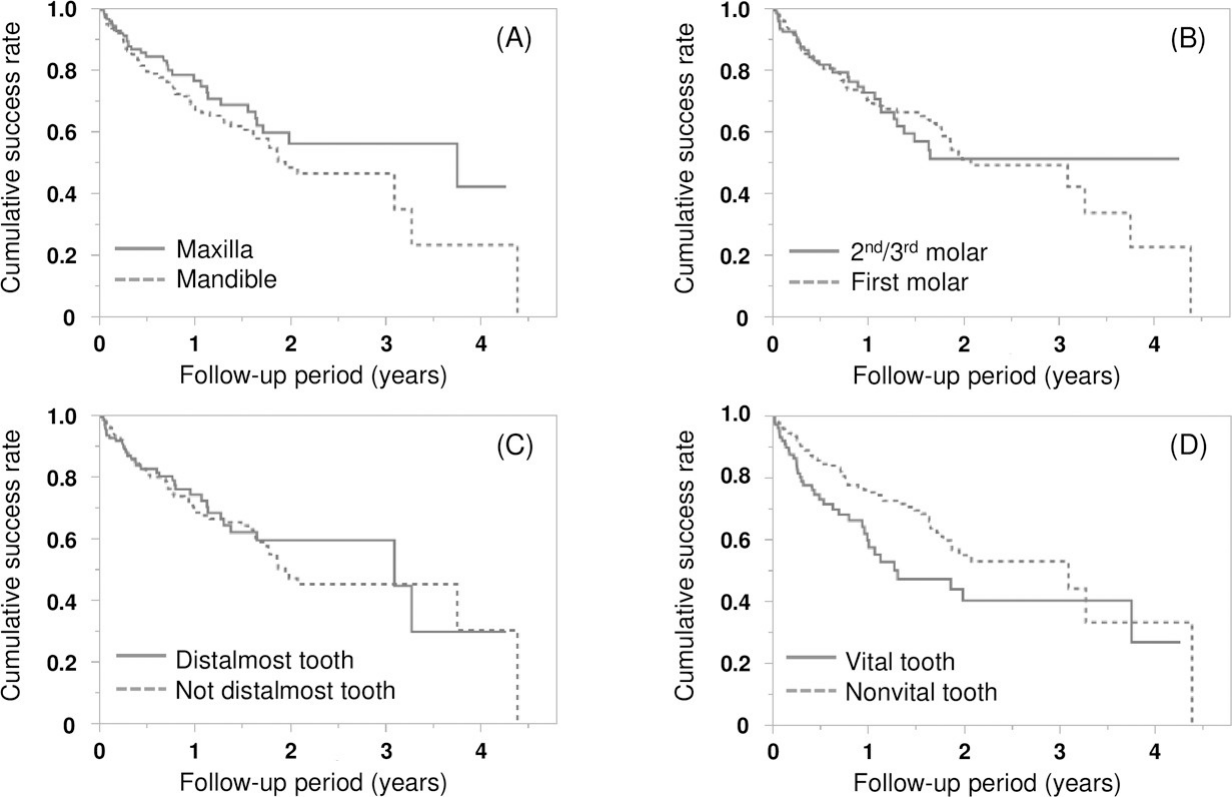
Kaplan–Meier curves for each covariate. Log-rank tests were performed for each covariate. (A) Maxilla/mandible (*P* = 0.170), (B) 2^nd^/3^rd^ molar and 1^st^ molar (*P* = 0.971), (C) distalmost tooth/not distalmost tooth (*P* = 0.568), and (D) vital tooth/nonvital tooth (*P* = 0.011).

**Table 2** shows the result of Cox proportional hazard analysis. The HRs for the factors related to the site of crown placement are listed in the table for model 1, which was established using sex and age as covariates. The HRs and 95% CIs of the covariates included in model 2 are as follows: mandible/maxilla, HR = 1.08 (95% CI; 0.70–1.68, *P* = 0.731); distalmost tooth/not distalmost tooth, HR = 0.86 (95% CI; 0.40–1.86, *P* = 0.699); and 1st molar/2nd or 3rd molar, HR = 0.87 (95% CI; 0.41–1.86, *P* = 0.715). There were no significant differences based on crown location in models 1 and 2, except for the vital/nonvital tooth in model 1 (HR = 1.55, 95% CI; 1.03–2.33, *P* = 0.034). **Fig 5** shows the success curves classified by each factor. The vital/nonvital tooth resulted in significant differences in the hazard analysis.

**Table 2:** Results of the influence of the location and vital/nonvital tooth on CAD/CAM composite crown complications, assessed using the Cox proportional hazard model (*n* = 362).

	Model 1		Model 2	
	HR (95% CI)	*P* value	HR (95% CI)	*P* value
Mandible (ref. maxilla)	1.23 (0.80–1.88)	0.342	1.08 (0.70–1.68)	0.731
1^st^ molar (ref. 2^nd^/3^rd^ molar)	0.81 (0.39–1.69)	0.570	0.87 (0.41–1.86)	0.715
Distalmost tooth (ref. not distalmost tooth)	0.78 (0.37–1.64)	0.510	0.86 (0.40–1.86)	0.699
Vital tooth (ref. nonvital tooth)	1.55 (1.03–2.33)	0.034	1.42 (0.93–2.15)	0.100

Model 1: Sex and age (≥ 65/< 65) were adjusted as covariates.

Model 2: The type of restorative material used on the antagonist teeth and the type of cement used (adhesive resin cement or other) were added as covariates to model 1.

HR: Hazard ratio; 95% CI: 95% Confidence interval.

## Discussion

In this study, 362 CAD/CAM composite crowns were followed for up to 1603 days (average 378 days, median 286 days). This is the first retrospective study to focus on the CAD/CAM composite crowns applied to molars alone. Several prospective studies have focused on molars; however, in these cases, the sample size was no more than 35 crowns [16]. Although the present study was a retrospective cohort study, the sample size was 10 times larger; therefore, the data obtained should be relatively reliable.

In this study, CAD/CAM composite crown treatments on mandibular first molars were the most common because these are covered by Japanese health insurance before other molar locations. The overall success rate was 70.7%, and the survival rate was 93.1%. The cumulative success and survival rates were 70.9% and 93.7%, respectively, after 1 year, 50.8% and 88.3%, respectively, after 2 years, and 49.5% and 86.5%, respectively, after 3 years based on the Kaplan–Meier curves. Approximately 30% of the patients had complications within 1 year, and about half of the patients had complications within 2 years. This result is comparable to the three-year results for CAD/CAM composite crowns (87.9% survival rate, 55.6% success rate) observed by Vanoorbeek [17]. In another previous study comparing nonmetal crowns, the three-year cumulative success rate of CAD/CAM crowns was reported to be less than 40% [18]. The HRs of complication occurrence for CAD/CAM crowns compared to feldspar ceramic crowns was 3.42. On the other hand, Sailer et al. reported the estimated five-year survival rate of metal–ceramic crowns to be 94.7% [19]. These results indicate that the success rate of CAD/CAM composite crowns is low compared to those of conventional crowns. Nonetheless, after the complication, many crowns can be used again, and the survival rate would be clinically appropriate. These observations are similar to the results of previous studies on premolars [12]. In particular, crown fracture on molars in the present study occurred in only 5 crowns out of 362 crowns (1.4%). This is almost the same percentage as observed for premolars (1.6%) [12], even though molars are subjected to stronger occlusal forces than premolars [13].

The stronger occlusal forces on molars than on premolars are the main reason why the mechanical properties of composite blocks for molars (compared to those of premolars) have been a focus for improvement by manufacturers. In particular, the criteria for CAD/CAM resin blocks for molar application are strictly determined by the Japan Dental Material Association (JDMAS 245:2017). Because the average bite force applied to molars is around 800 N [20,21], the resin blocks must contain more than 70% inorganic filler and have a 240 MPa three-point bending strength after storage in water at 37°C for 7 days. Based on our results, the performance of the current resin composite block products seems to be sufficient to resist the occlusal forces, resulting in few crown fractures.

In one comparative study, it was reported that all-ceramic crowns on the distalmost teeth showed significantly lower performance than those on the nondistalmost teeth [22]. Our study apparently contradicts the previous study because the statistical analysis showed no significant differences in crown complications between distalmost teeth and nondistalmost teeth. This discrepancy possibly resulted from differences in the material of the crown (ceramic versus composite resin), which affected the clinical success rates [17]. In addition, we applied the Cox proportional hazard model, in which the risks based on the differences in crown placement location (maxilla/mandible, distalmost tooth/not distalmost tooth, and first molar/second or third molar) and the tooth condition (vital/nonvital) were evaluated (**Table 2**). First, in model 1, age (≧ 65/< 65) and sex were included as covariates. In model 2, the cement type used (adhesive resin cement/other cement) and antagonist tooth characteristics (natural tooth, restored tooth, dental implant, missing tooth, and unknown) were added. The difference in the brand of the CAD/CAM resin block was not incorporated as a covariate because all products met certain flexural strength criteria of the material product regulation. In fact, a recent literature review has shown that the type of the commercially available CAD/CAM resin block materials does not influence the survival rate of CAD/CAM composite crown treatments [9]. In both models, there were no significant differences in the HR with respect to crown placement location. In addition, no differences in the success curves were observed when the success times for each site were compared using the Kaplan–Meier method and log-rank test (**Fig 5**). These results are consistent with those of the Cox proportional hazard model, which shows that CAD/CAM composite crowns could be clinically applied to any site in the molar region.

Although, model 1 showed a significant difference in the HR of 1.55 (95% CI: 1.03–2.33) for the vital/nonvital tooth, model 2 did not show a significant difference in HR. However, the HRs and 95% CIs were almost same as those of model 1, suggesting that increasing the number of covariates made correct detection difficult. In practice, because CAD/CAM composite crowns require a greater resin thickness than metal crowns, more dentin must be prepared (more than 1.5 mm of the occlusal reduction). This greater amount of preparation may affect the clinical outcomes. In particular, for vital teeth, the need for additional preparation might increase the possibility of pulpal symptoms. On the other hand, it is sometimes difficult to ensure sufficient thickness to avoid pulpitis or hypersensitivity, and, when the amount of reduction is insufficient, the thickness of the crown would also be insufficient, which could lead to fracture or deformation of the crown resulting in debonding. Although premolar CAD/CAM composite crowns as abutment teeth show no differences in clinical outcomes between vital and nonvital teeth [12,23], more attention should be paid in the preparation of molar crowns. Crucially, the anatomical differences in the teeth and pulp chamber may have influenced the results.

There was also a significant difference in HRs for the type of cement used, despite its being a covariate. Regarding cement type, the hazard ratio was 1.77 (95% CI: 1.17–2.68), suggesting that the cement could be related to the occurrence of complications. Because the type of cement used was highly correlated with the preparation facility, this is a limitation of the study design. In particular, in this study, the most common type of complication was crown debonding, which accounted for 74.5% of the complications. The importance of the adhesive procedure used for the placement of CAD/CAM composite crowns has been noted [24], and most of the debonding that occurs in the early stages after fitting is thought to be due to technical factors [25–27]. This trend can be seen in the relatively high rate of debonding in the period after fitting, as well as the fact that, after the debonded crowns were reattached, only 16% of the crowns (12 crowns) underwent subsequent debonding and the other crowns remained intact for a long time.

Because the elastic modulus of the CAD/CAM composite crowns is lower than that of ceramic crowns, the stress is concentrated in the cement layer and bonding interface [28]. Therefore, the compressive strength or the bond strength of the cement is more important for these crowns than those comprised of ceramic. Ankyu et al. demonstrated crown deformation during use compared to that of lithium disilicate ceramic crowns [29]. They found that CAD/CAM composite materials are easier to deform than ceramic materials. Further, molars might be more susceptible to cementation because of their larger surface area than premolars.

Because there were only two cases of RPD abutment teeth in this study, it was not possible to perform a statistical analysis for these cases. This low number might be a result of appropriate treatment selection because RPD abutment teeth are a risk factor for complications. In other words, if the CAD/CAM composite crowns were placed on RPD abutment teeth, the clinical performance could be expected to be poor.

As in the case of premolars, for the molars, problems were more frequent within the first 6 months than at later periods, although the complications continued to be observed for molars at a similar rate after 6 months (**Fig 3**). After 12 months, severe complications leading to tooth extraction or the removal of the crowns such as the fracture of the abutment teeth and crown fracture, which were rarely seen in the premolars, occurred, and these problems are suspected to be related to fatigue with time. Because the causes of these complications remain unknown, direct observation of the fractured crowns and mechanical investigation is considered necessary. As a result, a prospective clinical study should be conducted to evaluate the occlusal conditions and tooth contact habits of the patients, and the thickness and degree of occlusal wear of the crowns should be evaluated individually.

The limitations of this study include the fact that it was a retrospective cohort study, which makes it impossible to evaluate the actual morphology, crown thickness, and occlusal forces or the parafunction of each subject. Additionally, the investigation focused on patients from two facilities, which may have influenced the results because the method of impression, processing of the inner surface of the crown, and the type of cement used likely differ between facilities. The retrospective study design limited the acquisition of information on related parameters for virtual design during the manufacturing processes. Moreover, because there were several subjects having metal allergies, some of these subjects required the use of nonmetallic materials even on the vital molar tooth, which could have caused insufficient occlusal reduction.

Although the morphology of the abutment teeth was not evaluated in this study, Kabetani *et al*. [30] evaluated the axial surface height and crown thickness of abutment teeth in their study of premolar CAD/CAM composite crowns and reported that they were associated with axial surface height complications on the nonfunctional occlusal side. In addition, Yamaguchi *et al*. [31] has championed the use of artificial intelligence to predict the relationship between abutment tooth morphology and complications in CAD/CAM composite crown data for premolars. Therefore, with respect to molars, the abutment teeth should be morphologically evaluated to predict the clinical complications.

## Conclusions

Within the limitations of this study, the null hypothesis that differences in crown location would not affect the clinical outcomes of patients fitted with CAD/CAM composite crowns was not rejected, indicating that the location of CAD/CAM composite crowns is unlikely to be a risk factor for complications. Therefore, it is suggested that CAD/CAM composite crowns can be clinically applied to all molars. In addition, it is suggested that there might be a higher risk when crowns are applied to vital teeth, and the use of a cement other than adhesive resin cement might be associated with complications.
